# Sepsis Presentation, Interventions, and Outcome Differences Among Men and Women in the Emergency Department

**DOI:** 10.5811/westjem.40005

**Published:** 2025-07-11

**Authors:** Joseph O’Brien, Jon W. Schrock

**Affiliations:** * Cleveland Clinic Lerner College of Medicine, Department of Emergency Medicine, Cleveland, Ohio; † MetroHealth Medical Center, Department of Emergency Medicine, Cleveland, Ohio

## Abstract

**Objectives:**

Sepsis is a common presentation to the emergency department (ED) and represents a life-threatening syndrome with high mortality rates. The existing literature has conflicting findings regarding outcomes between sexes. Our goal in this study was to investigate the clinical presentation, interventions, and outcomes based on sex for sepsis in the ED.

**Methods:**

We conducted a retrospective cohort study to identify patients presenting with sepsis to the ED. We employed the Global Collaborative Network from 119 international healthcare organizations in the TriNetX Research Network. Sepsis was defined according to *International Classification of Diseases*, *10^th^ Rev*, codes. To evaluate sex differences in sepsis presentation, we collected data on age, comorbidities, sex, vital signs, laboratory values, medications, intensive care unit (ICU) admission, mechanical ventilation, and mortality at 30 days, 90 days, and one year. We used a 1:1 propensity score matching by age, race, comorbidities, and infection source to identify and balance potential risk factors across the study groups to investigate mortality, interventions, and intensive care unit admission trends. Data abstraction and analysis were conducted in the TriNetX platform.

**Results:**

In total, 920,160 patients were included in this study. The most common infection source for both females and males was respiratory, accounting for 40% and 46.2% of sepsis cases, respectively. After adjusting for urinary tract infection as an infection source, females were less likely to receive piperacillin-tazobactam (21% vs 23.6%; odds ratio [OR] 0.76; 95% confidence interval [CI] 0.75 – 0.77), vancomycin (32.9% vs 36%; OR, 0.87; 95% CI 0.86 – 0.88), and vasopressors (16.5% vs 17.6%; OR, 0.92; 95% CI 0.91 – 0.93). Females had a lower all-cause mortality at 30 days (12.1% vs 13%; OR 0.91; 95% CI 0.90 – 0.92), 90 days (17.1% vs 18.7%; OR 0.91; 95% CI 0.90 – 0.92), and one year (21.5% vs 23.3%; OR 0.90; 95% CI 0.89 – 0.91).

**Conclusion:**

Females demonstrated 10% lower odds of mortality from sepsis at 30 days, 90 days, and one year (absolute difference: 0.9%, 1.6%, 1.8%, respectively). Females were less likely to receive vasopressors, vancomycin, or piperacillin-tazobactam, even after accounting for urinary tract infection as the sepsis source.

## INTRODUCTION

Sepsis is a common presentation to the emergency department (ED) that if not promptly recognized and treated can lead to severe end-organ damage, coma, and death. Despite decades of research efforts, mortality from sepsis remains high, with one meta-analysis on European, North American, and Australian patients reporting an average 30-day mortality of 24.4%.^1^ Although the causes of sepsis are multifactorial, one proposed area of research for furthering understanding and treatment of sepsis is differences related to sex. Worldwide, there has been a growing understanding of the necessity for research—from basic science to clinical studies—to collect, stratify, and analyze data based on sex. This is an essential area of research, as females have been historically under-represented in clinical trials, and sex-dependent differences affecting disease pathogenesis, outcomes, and response to treatment have been consistently reported.^2^

Men and women have been reported to have different immune responses to infections, likely due to genetic, hormonal, epigenetic, and environmental factors.^3^ The exact mechanism by which sex differences affect the initial clinical presentation and outcomes of sepsis in the ED has yet to be fully elucidated. There are conflicting reports in the literature, with some studies finding no difference^4^,^5^ and others showing higher mortality in women,^6^ while yet others found higher mortality in men.^7^ A meta-analysis of >25,000 pooled patients found females had a slightly higher mortality than males, although two of the largest cohorts included in the analysis reported opposing findings, rendering the overall meta-analysis inconclusive.^8^–^10^ A large retrospective study in Australia reported that among older adults, males had significantly higher sepsis mortality, intensive care unit (ICU) admission, and hospital readmission.^11^,^12^ Most of these studies have been limited to patients in the ICU, with fewer studies looking at the role of sex in the initial presentation to the ED.^13^,^14^

Several studies have demonstrated that female patients experience delays in receiving antibiotics, a critical component of sepsis management. Retrospective cohort analyses found that women presenting with sepsis or septic shock had significantly longer median time-to-antibiotics compared to men, even after adjusting for confounding factors such as infection source and severity scores.^15^,^16^ These delays likely contribute to clinical deterioration, potentially negating any physiological advantages associated with female immune responses. Furthermore, treatment disparities extend beyond antibiotic administration. For instance, systematic reviews and observational studies highlight that females are less likely to receive aggressive resuscitation with fluids, timely vasopressor therapy, or organ support despite similar or worse sepsis severity.^17^,^18^

Beyond treatment delays, differences in clinical presentation between sexes may contribute to diagnostic challenges. Studies have identified that female sepsis patients often exhibit atypical symptoms such as fatigue or altered mental status rather than classic indicators such as fever and tachycardia, more commonly seen in males.^17^,^19^ This variation in symptomatology may lead to under-recognition, delays in triage prioritization, and altered clinical decision-making. Early detection and initiation of treatment for sepsis is critical for improving patient outcomes and decreasing mortality.

Given these discrepancies, we recognized a need to further investigate sex differences among the initial patient presentation to the ED of the septic patient. In this study we aimed to address these gaps in the literature through a retrospective cohort study using a multicenter research network investigating the association between patient sex and clinical presentation with clinical outcomes.

**Population Health Research Capsule**

What do we already know about this issue?

*Sepsis is a frequently encountered condition in the ED with high mortality. Existing literature presents conflicting evidence regarding outcome differences between sexes*.

What was the research question?

*Are there differences based on sex on the clinical presentation, interventions, and outcomes of sepsis in the ED?*

What was the major finding of the study?

*Females had lower mortality at 30- (12.1% vs 13%; OR 0.91; 95% CI 0.90*–*0.92), and 90 days (17.1% vs 18.7%; OR 0.91; 95% CI 0.90*–*0.92), and at one year (21.5% vs 23.3%; OR 0.90; 95% CI 0.89*–*0.91)*.

How does this improve population health?

*Recognizing sex-based differences in sepsis interventions and outcomes may guide more equitable treatment strategies and improve survival rates across populations*.

## METHODS

### Study Design and Data Source

We conducted a large, retrospective, cohort study to identify patients presenting with sepsis to the ED. We employed the Global Collaborative Network from 119 healthcare organizations (HCO) in the TriNetX Research Network. TriNetX is a federated research network encompassing over 100 HCOs across the world. It facilitates real-time access to healthcare records, featuring de-identified data from more than 250 million patients across various HCOs. The data is sourced directly from the electronic health record management systems (EHR) of participating organizations, which range from large academic centers providing tertiary care to satellite outpatient office locations.

Clinical variables are derived from clinical documents using a built-in natural language processing system, ensuring that robust quality assurance procedures are implemented prior to inclusion in the database. TriNetX safeguards patient privacy by offering only aggregate counts and statistical summaries, maintaining de-identification of data throughout the retrieval and dissemination processes.

### Study Definitions, Variables, and Outcomes

The available data included information about the demographics, diagnoses (based on the *International Classification of Diseases, 10^th^ Rev, Clinical Modification*, [ICD-10-CM] codes and procedures coded in the ICD-10 Procedure Coding System or Current Procedural Terminology); medications coded in the Veterans Affairs National Formulary; laboratory tests coded in Logical Observation Identifiers Names and Codes and healthcare utilization. Sepsis was defined according to the ICD-10-CM diagnosis code. Any adult (18 years of age) who presented to and was diagnosed with sepsis in the ED in the Global Collaborative Network from the HCOs within the TriNetX system were included. We excluded minors and pregnant patients from the study.

To evaluate sex differences in the sepsis presentation, we collected the following variables: age; sex; vital signs; white blood count; lactate; hemoglobin; platelets; ICU admission; antibiotics use; intravenous fluids, mechanical ventilation; infection source; and 30-day, 90-day, and one-year mortality. Intervention data, such as the administered medication, was collected if the medication was given within 24 hours of the patient’s presentation to the ED.

### Statistical Analysis

The TriNetX platform uses input matrices containing user-identified covariates and employs logistic regression analysis to derive propensity scores for individual subjects. Subsequently, 1:1 matching is conducted based on these propensity scores, using greedy nearest neighbor algorithms with a caliper width of 0.1 pooled standard deviations. To mitigate bias resulting from these algorithms, TriNetX randomizes the order of rows. Through this process, individuals with similar propensities, and hence similar comorbidity profiles, were matched, minimizing potential confounding effects and allowing for a more balanced comparison between male and female cohorts in terms of their comorbidities and other potential confounders. This study method has been previously validated.^12^,^20^–^22^ Missing data in the TriNetX network was addressed using median imputation, where the median value of the specific variable was used to replace missing entries.

This study followed several elements of an optimal retrospective chart review, as detailed by Worster and Bledsoe.^23^ We designed the study methodology to ensure rigor and consistency. Data collection was conducted using the TriNetX platform, which automates extraction from EHRs through natural language processing. Consequently, we did not use manual abstractors, eliminating the need for abstractor training, data abstraction forms, performance monitoring, or interobserver reliability testing. Inclusion and exclusion criteria for sepsis cases were clearly defined based on ICD-10-CM codes, and key variables, such as demographics, vital signs, laboratory values, interventions, and outcomes, were precisely described. The TriNetX platform employs robust data quality-assurance procedures, ensuring consistent and reliable data across all participating HCOs. Because the dataset was de-identified and retrospective, the risk of abstractor bias was inherently mitigated. We included in the analysis all patients meeting the inclusion criteria, ensuring comprehensive sampling. Missing data were managed using median imputation for the specific variables, maintaining dataset integrity.

## RESULTS

### Study Population

In total, 1,003,928 patients were included in this study: 47.4% females, and 52.6% males (Table 1). Most patients in both cohorts were White. Males and females who presented to the ED with sepsis had similar rates of diabetes, cerebrovascular disease, liver disease, peptic ulcer disease, chronic obstructive pulmonary disease, heart failure, peripheral vascular disease, cerebrovascular disease, and neoplasms. A significantly greater proportion of males presented with a history of ischemic cardiac disease compared to their female counterparts (26.6% vs 20.9%; *P* < .0001). Males also presented with a significantly greater proportion of chronic kidney disease compared to females (20.1% vs 18.2%; *P* < .0001). In both cohorts, a history of neoplasms and diabetes were the two most common comorbidities.

### Clinical Presentation

All the covariates used for matching in the two groups were similar after propensity score matching (mean standard difference <0.1) (Table 1). Males and females with sepsis presented to the ED with similar initial vital signs (Table 2). While there was a statistically significant difference between systolic blood pressure, diastolic blood pressure, respiratory rate, and heart rate, these differences were probably not clinically remarkable. Notably, a significantly greater proportion of males (39.1% vs 36.4%) presented with a fever >100°F compared to females (*P* < .0001). Males and females had a similar degree of leukocytosis, with females presenting with significantly greater platelet count than men (*P* < .0001). Females also demonstrated significantly lower lactate levels compared to men (*P* < .0001).

### Clinical Interventions

Among both males and females, penicillin was the most commonly administered antibiotic within 24 hours of presentation to the ED (Table 2). Females were less likely to receive vasopressors (OR 0.87; 95% CI 0.86 – 0.89), beta-lactamase inhibitors (OR 0.84; 95% CI, 0.83 – 0.85), first-generation cephalosporins (OR 0.84; 95% CI, 0.83 – 0.86), fourth-generation cephalosporins (OR 0.95; 95% CI, 0.94 – 0.96), clindamycin (OR 0.91; 95% CI, 0.89 – 0.93), macrolides (OR 0.92; 95% CI 0.91 – 0.93), sulfonamides (OR 0.92; 95% CI 0.91 – 0.94), and vancomycin (OR 0.85; 95% CI 0.84 – 0.86). Females were more likely to receive second-generation cephalosporins (OR 1.29; 95% CI 1.24 – 1.34), third-generation cephalosporins (OR 1.06; 95% CI 1.05 – 1.07), quinolones (OR 1.17; 95% CI 1.16 – 1.19), metronidazole (OR 1.12; 95% CI 1.10 – 1.14), and carbapenems (OR 1.14; 95% CI 1.11–1.16).

Ceftriaxone and piperacillin-tazobactam are two commonly prescribed antibiotics used in the sepsis treatment pathway. Females were less likely to receive piperacillin-tazobactam (OR 0.85; 95% CI 0.84 – 0.86) than males who presented to the ED. However, females were more likely to receive ceftriaxone (OR 1.23; 95% CI 1.22 – 1.24) than males.

### Clinical Outcomes

We used propensity score matching to establish comparable cohorts with respect to morbidities between male and females. The most common infection source for both females and males was respiratory, accounting for 40% and 46.2% of sepsis cases, respectively (Table 3). A urinary infection source was found in 29.7% of females and 18.7% of males. A urinary infection source was more common in females (OR [odds ratio] 1.73; 95% confidence interval [CI] 1.71 – 1.75), whereas respiratory, abdominal, and skin and soft tissue infection sources were more common in males (Table 3). Approximately two-thirds of both cohorts were admitted to the hospital. Septic shock occurred in 15.2% of females and 15.6% of males. The all-cause mortality rate at 90 days was 17.1% for females and 18.7% for males (Figure). Females had a lower all-cause mortality rate at 30 days (OR 0.91; 95% CI 0.90 – 0.92); 90 days (OR 0.91; 95% CI 0.90 – 0.92); and one year (OR 0.90; 95% CI 0.89 – 0.91). Females were also less likely to require ICU admission within 30 days (OR 0.85; 95% CI 0.84 – 0.86) or mechanical ventilation (OR 0.76; 95% CI 0.75 – 0.77).

We found that urinary infection sources were more common in females than males, and previous studies have shown urinary tract infections (UTI) have better clinical outcomes and reduced mortality compared to other infection sources.^24^ We performed a secondary analysis in which UTIs were included in the 1:1 propensity score matching to account for this potential confounder (Supplemental Table 1). Females were still less likely to receive piperacillin-tazobactam (OR 0.76; 95% CI 0.75 – 0.77), vancomycin (OR 0.87; 95% CI 0.86 – 0.88), and vasopressors (OR 0.92; 95% CI 0.91 – 0.93). Females also had a lower all-cause mortality at 30 days (OR 0.94; 95% CI 0.93 – 0.95); 90 days (OR, 0.95; 95% CI 0.94 – 0.96); and one year (OR 0.93; 95% CI 0.92 – 0.94).

## DISCUSSION

This multicenter, retrospective, cohort study is one of the largest studies investigating the ED clinical presentation and outcomes of sepsis based on sex. We report several differences in initial presentation and outcome of sepsis between the sexes. Males were more likely to have chronic kidney disease or ischemic heart disease than their female counterparts, but otherwise the two cohorts had similar rates of comorbidities. Males also had a higher frequency of fever, similar to what was reported in other published studies.^14^ While the differences between the presenting heart rate, systolic blood pressure, diastolic blood pressure, and respiratory rates were statistically significantly between the sexes, the differences were not clinically remarkable. Females tended to have a greater platelet count and lower serum lactate than males, in line with the published literature.^14^ Platelets play a dual role, not only participating in coagulation activation but also contributing to the acute phase response in infectious diseases and enhancing innate immune cell responses.^25^ Thrombocytopenia frequently occurs in sepsis and correlates with organ failure, a reduced immune function, and a poor prognosis.^26^ Females were also more likely to present with a UTI compared to males, a finding that has been reported in other studies.^4^,^27^ Males were more likely to have a respiratory, abdominal, skin and soft tissue infection, or unknown cause as the source of their infection.

This is one of the first studies to report on medication treatment patterns in the ED for sepsis based on sex. We report that females were more likely to receive second- and third-generation cephalosporins, quinolones, metronidazole, and carbapenems compared to males. Females were less likely to receive vasopressors, beta-lactamase inhibitors, first- and fourth-generation cephalosporins, clindamycin, macrolides, sulfonamides, and vancomycin. Ceftriaxone and piperacillin-tazobactam are two broad-spectrum antibiotics that are commonly prescribed to any patient suspected of sepsis. We found that females were less likely to receive piperacillin-tazobactam, but they were more likely to receive ceftriaxone. This difference likely reflects that UTIs were more commonly the infection source in females compared to males. To account for this difference, we performed a secondary analysis in which UTI was included in the propensity score matching criteria to create two balanced cohorts with approximately equal numbers of patients with UTIs. After adjusting for this potential confounder, females were still less likely to receive vasopressors, vancomycin, or piperacillin-tazobactam. Females also had a lower rate of in-hospital mortality.

We report that females had 10% lower odds of mortality at 30 days, 90 days, and one year. There are conflicting reports in the literature regarding mortality differences between males and females for sepsis. Some studies have reported that males have increased mortality,^7^,^28^ whereas others have found that females have increased mortality,^29^,^30^ and yet others have found no difference.^14^,^31^ Some of these studies had smaller sample sizes and may have been underpowered to detect a mortality difference.^14^ We employed propensity score matching to demonstrate that females exhibit diminished odds of ICU admission, requirement for mechanical ventilation, and mortality, thereby revealing sex-based differences in critical care outcomes.

The difference in mortality between males and females may be due to differences in sex steroid levels. Estrogens are known to influence immune functions, although the precise mechanisms remain incompletely understood. They primarily regulate innate immune function by suppressing the activation of monocyte-macrophage cells and reducing the production of inflammatory cytokines.^32^ Additionally, estrogens impact neutrophil behavior, enhance dendritic cell maturation, and increase TH1 and TH17 activity.^32^ Estrogens are suggested to confer a protective influence in sepsis by modulating the release of inflammatory cytokines and inhibiting tissue neutrophil infiltration, thereby mitigating organ damage.^33^ Additionally, estrogen shields against sepsis-induced liver injury by modulating mitochondrial function and activating inflammatory-mediated pyroptosis signaling pathways.^34^

The strengths of this study are its multicenter design that captured a large patient sample across 119 HCOs from around the world. The large sample size powered the study to detect differences in clinical presentations and outcomes of sepsis between the sexes. Given the global distribution of the participating HCOs in the TriNetX database, this study has good generalizability. However, there are several limitations.

## LIMITATIONS

Because we used a large international database that lacked line-level data, we were unable to determine sepsis severity as determined through quick Sequential Organ Failure Assessment or severe inflammatory response syndrome criteria at the patient level. This could potentially have introduced confounding and should be further investigated in future studies. Furthermore, patient outcomes and practice patterns may differ based on location and year. Due to the lack of line-level data provided in the TriNetX network, we were unable to control for these potential confounders. Another limitation of our study was the reliance on ICD-10 codes to define sepsis, which may have potentially missed some cases due to coding inaccuracies or variations in coding practices across different healthcare settings and regions. Reliance on a sepsis diagnosis based on ICD-10 code upon discharge from the ED may have missed patients who were diagnosed with sepsis later upon their admission to the hospital. Despite these potential shortcomings, this study was among the largest to investigate clinical presentation, interventions, and outcome differences in sepsis based on sex.

## CONCLUSION

In this large retrospective cohort study, we found that females with sepsis had lower mortality rates at 30 days, 90 days, and one year compared to males, despite receiving fewer vasopressors or certain broad-spectrum antibiotics like vancomycin or piperacillin-tazobactam. While these findings suggest potential sex-based differences in sepsis management and outcomes, the underlying mechanisms remain unclear. Further research is needed to explore biological, clinical, and systemic factors that may contribute to these differences and to assess how treatment disparities impact patient outcomes.

## Supplementary Information



## Figures and Tables

**Figure:**
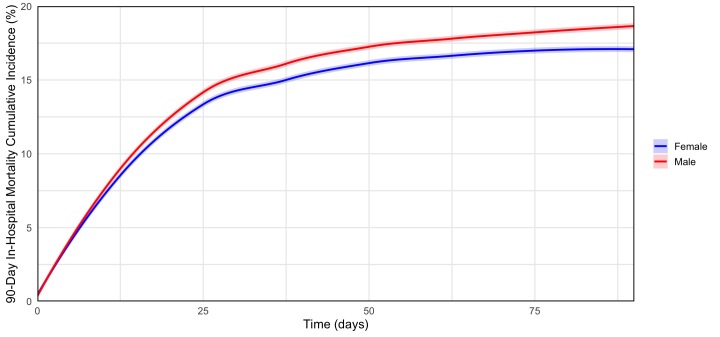
Cumulative incidence of in-hospital mortality for patients presenting to the emergency department, stratified by sex. Males had a greater in-hospital mortality at 90 days compared to females (HR: 0.91; 95% CI 0.90–0.92; *P*<0.0001). Propensity score matching was used to ensure comparable distributions of comorbidities between the male and female cohorts. Ribbons depict 95% confidence interval.

**Table 1:** Characteristics of men and women who present to the emergency department with sepsis.

	Before matching			After matching		
	
	Females–n (%) n = 475,812	Males–n(%) n = 528,116	SD	Females–n (%) n = 460,080	Males–n(%) n = 460,080	SD
Demographics						
Age at Index (years)	60.5± 21.3	60.4 ± 19.7	0.004	60.1± 21.4	60.3 ± 20.7	0.011
Race						
White	301,623 (63.7)	340,824 (64.9)	0.024	282,524 (63.5)	291,401 (65.1)	0.032
Black	71,731 (15.2)	73,032 (13.9)	0.036	68,133 (15.2)	60,248 (13.5)	0.05
Other	52,988 (11.2)	57,497 (10.9)	0.008	50,856 (11.4)	50,204 (11.2)	0.005
Comorbidities						
Neoplasms	151,338 (32.2)	161,917 (30.8)	0.02	137,654 (30.7)	141,705 (31.6)	0.02
Diabetes	136,938 (28.0)	155,305 (28.7)	0.015	126,293 (27.3)	128,126 (27.1)	0.009
Ischemic cardiac disease	101,981 (20.9)	144,009 (26.6)	0.14	92,760 (20.1)	111,001 (24.0)	0.09
Chronic kidney disease	89,083 (18.2)	108,854 (20.1)	0.048	82,027 (17.7)	83,769 (18.1)	0.010
Heart failure	86,413 (17.7)	98,893 (18.3)	0.015	75,066 (16.2)	75,331 (16.3)	0.002
Peripheral vascular disease	36,014 (7.4)	45,498 (8.4)	0.04	31,720 (6.9)	31,390 (6.8)	0.0003
Cerebrovascular disease	33,741 (6.9)	37,124 (6.9)	0.0018	30,242 (6.5)	30,453 (6.6)	0.0018
Liver disease	15,301 (3.1)	17,908 (3.3)	0.01	13,830 (3.0)	14,210 (3.1)	0.0005
Peptic ulcer disease	8,116 (1.7)	7,878 (1.5)	0.017	6,714 (1.5)	6,765 (1.5)	0.0001
Chronic obstructive pulmonary disease	71,177 (14.6)	76,037 (14.0)	0.015	62,540 (13.5)	63,550 (13.7)	0.006

Categorical variables are given in total number of patients and valid percentages (%). Continuous variables are depicted as mean ± SD.

**Table 2:** Clinical presentation and interventions performed in the emergency department stratified by sex.

Characteristic	Females–n (%) N = 460,080	Males–n(%) N = 460,080	OR	95% CI	*P*-value
ED Presentation					
Fever > 100°F	183,959 (36.4)	218,089 (39.1)	—	—	<.0001
SBP (mmHg)	106.6 ± 23.9	108.2 ± 23.9	—	—	<.0001
DBP (mmHg)	59.0 ± 13.7	61.7 ± 13.8	—	—	<.0001
HR (bpm)	86 ± 18.8	84.3 ± 19.2	—	—	<.0001
RR (breathes/min)	18.2 ± 3.82	18.4 ± 3.91	—	—	<.0001
Altered Mental Status	54,749(11.9)	54,289(11.8)	—	—	<.0001
Laboratory findings					
WBC / uL	17.1 ± 7.7	17.4 ± 7.9	—	—	<.0001
Platelets / uL	260 ± 116	236 ± 115	—	—	<.0001
Hemoglobin (g/dL)	11.6 ± 2.13	12.2 ± 2.5	—	—	<.0001
Lactate (mmol/L)	1.91 ± 1.81	1.99 ± 1.79	—	—	<.0001
IV fluids	369,190 (80.2)	370,071 (80.4)	0.99	0.98–1.01	.07
Vasopressors	75,000 (16.3)	84,019 (18.2)	0.87	0.86–0.89	<.0001
Antibiotics					
Penicillins	301,593 (65.5)	300,504 (65.3)	1.01	1.0–1.02	.0208
Beta-lactamase inhibitors	101,905 (22.1)	116,260 (25.2)	0.84	0.83–0.85	<.0001
Piperacillin-tazobactam	94,998 (20.6)	107,398 (23.3)	0.85	0.84–0.86	<.0001
Cephalosporins					
1st generation	22,491 (4.9)	26,414 (5.7)	0.84	0.83–0.86	<.0001
2nd generation	5,268 (1.1)	4,105 (0.9)	1.29	1.24–1.34	<.0001
3rd generation	216,323 (47.0)	460,080 (45.6)	1.06	1.05–1.07	<.0001
Ceftriaxone	140,267 (30.5)	121,087 (26.3)	1.23	1.22–1.24	<.0001
4th generation	80,110 (17.4)	83,303 (18.1)	0.95	0.94–0.96	<.0001
Quinolones	52,865 (11.5)	45,901 (9.9)	1.17	1.16–1.19	<.0001
Clindamycin	17,275 (3.8)	18,926 (4.1)	0.91	0.89–0.93	<.0001
Macrolides	57,174 (12.5)	61,462 (13.4)	0.92	0.91–0.93	<.0001
Sulfonamides	9,178 (2.0)	9,781 (2.2)	0.94	0.91–0.94	<.0001
Vancomycin	149,574 (32.5)	165,948 (36.1)	0.85	0.84–0.86	<.0001
Metronidazole	51,211 (11.1)	46,031 (10.0)	1.12	1.10–1.14	<.0001
Tetracycline	20,544 (4.5)	20,772 (4.5)	1.0	0.99–1.01	0.25
Carbapenem	23,776 (5.1)	20,997 (4.6)	1.14	1.11–1.16	<.0001

Medication administration data were collected within 24 hours of initial presentation to the ED. Propensity score matching was employed to ensure comparable distributions of comorbidities between the male and female cohorts. Categorical variables given in total number of patients and valid percentages (%). Continuous variables are depicted as mean ±SD.

*OR*, odds ratio; *CI*, confidence interval; *ED*, emergency department; *DBP*, diastolic blood pressure; *mmHg, millimeters of mercury* ; *HR*, heart rate; *bpm*, beats per minute; *RR*, respiration rate; SBP, systolic blood pressure; *WBC*, white blood cell count; *IV*, intravenous.

**Table 3:** Outcomes of sepsis patients stratified by sex.

Outcome	Females–n (%) N = 460,080	Males–n (%) N = 460,080	OR	95% CI	P-value
Mechanical ventilation	47,848 (10.4)	67,172 (14.6)	0.76	0.75 – 0.77	<.0001
ICU admission within 30 days	135,263 (29.4)	167,929 (36.5)	0.85	0.84 – 0.86	<.0001
Infection source					
Respiratory	184,032 (40.0)	212,557 (46.2)	0.86	0.85 – 0.87	<.0001
Urinary	136,643 (29.7)	86,034 (18.7)	1.73	1.71 – 1.75	<.0001
Abdominal	17,022 (3.7)	14,722 (3.2)	1.15	1.12 – 1.17	<.0001
Skin and soft tissue	47,388 (10.3)	63,491 (13.8)	0.72	0.71 – 0.73	<.0001
Unknown origin	74,993 (16.3)	83,274 (18.1)	0.74	0.73 – 0.75	<.0001
Septic shock	69,932 (15.2)	71,772 (15.6)	0.97	0.96 – 0.98	<.0001
Hospital Admission	308,253 (67.2)	311,014 (67.6)	0.98	0.97 – 0.99	<.0001
In-hospital mortality					
30-day mortality	55,670 (12.1)	59,810 (13.0)	0.91	0.90 – 0.92	<.0001
90-day mortality	78,673 (17.1)	86,034 (18.7)	0.91	0.90 – 0.92	<.0001
1-year mortality	98,917 (21.5)	107,199 (23.3)	0.90	0.89 – 0.91	<.0001

Propensity score matching was employed to ensure comparable distributions of comorbidities between the male and female cohorts.

*OR*, odds ratio; *CI*, confidence interval; ICU, intensive care unit.
