# Supplementary material for: Sepsis Presentation, Interventions, and Outcome Differences Among Men and Women in the Emergency Department
Source: West J Emerg Med. 2025 Jul 11;26(4):880–7. doi: 10.5811/westjem.40005 (PMC12342512; doi:10.5811/westjem.40005)
Supplement: Supplementary file 1 [file wjem-26-880-s001.docx]

Supplemental Table 1: Interventions and outcomes of sepsis patients, stratified by sex and adjusted for urinary tract infection as sepsis source. Medication administration data were collected within 24 hours of initial presentation to the Emergency Department. Propensity score matching was employed to ensure comparable distributions of comorbidities between the male and female cohorts. Categorical variables given in total number of patients and valid percentages (%). Continuous variables are depicted as mean plus/minus the standard deviation.

| Characteristic | Females–no. (%)  n = 440,366 | Males–no.(%)  n = 440,366 | OR | 95% CI | P-value |
| --- | --- | --- | --- | --- | --- |
| Medications |  |  |  |  |  |
| IV Fluids | 369,026 (83.8) | 369,907 (84.0) | 0.99 | 0.98—1.01 | 0.09 |
| Vasopressors | 72,694 (16.5) | 7,525 (17.6) | 0.92 | 0.91–0.93 | <0.0001 |
| Antibiotics |  |  |  |  |  |
| Penicillins | 281,249 (63.9) | 283,794 (64.5) | 0.98 | 0.96—1.0 | 0.041 |
| Beta-lactamase inhibitors | 99,488 (22.6) | 112,545 (25.6) | 0.85 | 0.84–0.86 | <0.0001 |
| Piperacillin-tazobactam | 92,722 (21.0) | 104,062 (23.6) | 0.86 | 0.85–0.87 | <0.0001 |
| Cephalosporins |  |  |  |  |  |
| 1st generation | 21,842 (5.0) | 25,535 (5.8) | 0.85 | 0.83–0.86 | <0.0001 |
| 2nd generation | 5,290 (1.2) | 4,200 (0.9) | 1.26 | 1.24–1.34 | <0.0001 |
| 3rd generation | 204,782 (46.5) | 203,767 (46.2) | 1.0 | 0.99—1.01 | 0.06 |
| Ceftriaxone | 130,120 (29.6) | 118,608 (26.9) | 1.14 | 1.13—1.15 | <0.0001 |
| 4th generation | 75,276 (17.1) | 77,124 (17.5) | 0.97 | 0.96—0.98 | <0.0001 |
| Quinolones | 51,562 (11.7) | 46.366 (10.5) | 1.13 | 1.11–1.15 | <0.0001 |
| Clindamycin | 16,966 (3.9) | 17.914 (4.1) | 0.94 | 0.92—0.96 | <0.0001 |
| Macrolides | 56,723 (12.9) | 58,943 (13.4) | 0.95 | 0.94—0.96 | <0.0001 |
| Sulfonamides | 9,131 (2.1) | 9,699 (2.2) | 0.94 | 0.91–0.97 | <0.0001 |
| Vancomycin | 145,087 (32.9) | 158,406 (36.0) | 0.87 | 0.86—0.88 | <0.0001 |
| Metronidazole | 49,591 (11.3) | 43,107 (9.8) | 1.17 | 1.15–1.19 | <0.0001 |
| Tetracycline | 20,501 (4.7) | 19,719 (4.5) | 1.0 | 0.99–1.01 | 0.12 |
| Carbapenem | 22,965 (5.2) | 21, 221 (4.8) | 1.09 | 1.07–1.11 | <0.0001 |
| Outcomes |  |  |  |  |  |
| In-hospital mortality |  |  |  |  |  |
| 30-day mortality | 59,889 (13.6) | 62,972 (14.3) | 0.94 | 0.93–0.95 | <0.0001 |
| 90-day mortality | 76,623 (17.4) | 80,146 (18.2) | 0.95 | 0.94–0.96 | <0.0001 |
| 1-year mortality | 99,522 (22.6) | 104,807 (23.8) | 0.93 | 0.92–0.94 | <0.0001 |
